# Preparation and Product Characterization of Microwaveable Food Using *Lentinus edodes* Protein through 3D Printing

**DOI:** 10.3390/polym15183736

**Published:** 2023-09-12

**Authors:** Na Li, Hongbo Li, Zhenbin Liu, Shuang Lv, Suya Xie, Chunyang Shi, Yue Wu

**Affiliations:** 1 College of Food Science and Engineering, Central South University of Forestry and Technology, Changsha 410004, China; 20200100082@csuft.edu.cn; 2 School of Food Science and Engineering, Shaanxi University of Science and Technology, Xi’an 710021, China; hongbo715@163.com (H.L.); zhenbinliu@sust.edu.cn (Z.L.); lvshuang0225@sust.edu.cn (S.L.); xiesuya111@163.com (S.X.); shichunyang@sust.edu.cn (C.S.)

**Keywords:** *Lentinus edodes* protein, potato flour, xanthan gum, 3D printing, microwaveable food

## Abstract

The *Lentinus edodes* protein (LP) is a high-quality protein known for its well-balanced amino acid composition. In this study, we developed three-dimensional (3D)-printed microwaveable food using a combination of LP and potato flour, and optimized the formulation to achieve a ratio of LP: potato flour: xanthan gum: water = 2:8:1:23. The 3D-printed samples exhibited better shape, weight, and size compared to the molded samples after microwave treatment, with the most favorable microwave effect observed at a 90% filling ratio. The LP content affected the viscosity and retrogradation value of the LP–potato starch mixture. Microwave duration affected the surface hardness, interior softness, and moisture content of the product. The highest overall score of 8.295 points was obtained with a microwave processing duration of 2 min. This study lays a foundation for the development of LP-based 3D-printed food.

## 1. Introduction

The development of three-dimensional (3D) printing technology has demonstrated significant potential in various fields [1]. Within the food industry, 3D printing has emerged as a promising technique for creating intricate and customized food products [2]. A crucial aspect of 3D printing for food involves the advancement of edible ink, facilitating the creation of food products with diverse shapes, textures, and flavors [3]. Potatoes, a versatile ingredient in the food industry, have been studied as a potential substrate for edible ink in 3D printing [4]. However, the use of potatoes in 3D printing edible ink presents certain limitations, primarily due to their inadequate rheological properties, which pose challenges during the printing process [5]. To overcome these limitations, it becomes necessary to enhance the rheological properties of potato ink by incorporating high-quality proteins or other components. The *Pleurotus ostreatus* protein is one such protein that has been investigated for its potential in improving the rheological properties of 3D printing ink [6]. The *Lentinus edodes* protein (LP) is renowned for its rich and well-balanced amino acid composition, but its rheological gel properties have not been reported [7,8]. We anticipate that LP will not only enhance the nutritional value of potato powder, but also improve the printability of potato ink, thereby facilitating the creation of high-quality 3D-printed food.

Microwave technology has been widely applied in the food industry for various purposes, including heating, drying, and sterilization, owing to its unique advantages [9]. In the realm of 3D printing for food, microwave processing has gained attention for its potential benefits as well [10]. Microwave-assisted 3D printing offers advantages such as rapid heating, uniform energy distribution, and precise temperature control [11]. The integration of microwave technology into the 3D printing process can enhance the efficiency of food production, improve the quality of printed food items, and enable the creation of intricate structures with enhanced functionality [12,13]. Numerous studies have explored the application of microwave-assisted 3D printing in diverse food scenarios, including the printing of chocolate, meat products, and bakery goods [13,14,15,16]. These applications demonstrate the versatility and potential of microwave-assisted 3D printing for food in the development of novel food products and the optimization of food processing techniques, thereby meeting the evolving demands of consumers.

The aim of this study was to investigate the feasibility of utilizing LP and potato powder as raw materials for microwaveable 3D-printed food. The rheological properties and 3D printing performance of the raw materials were analyzed to optimize the printing performance of the edible ink in 3D printing [17]. By carefully adjusting the microwave processing duration, the physicochemical properties and sensory evaluation of the resulting food products were assessed. Analyzing the rheological properties of the LP and potato powder mixture provided valuable insights into the printing performance of the 3D edible ink. The findings of this study provided a theoretical foundation for the development and application of LP-based instant food. By leveraging the potential of 3D printing technology and optimizing the parameters of microwave processing, it becomes possible to prepare high-nutrition and great-tasting microwave food, accompanied with improved texture and sensory attributes. This research contributes to the advancement of the field and unlocks new prospects for the creation of innovative and convenient food products.

## 2. Materials and Methods

### 2.1. Materials and Instruments

LP (purity 90%) was provided by Qingzhi Biological Technology Co., Ltd., Xi’an, China. Xanthan gum (XG, food grade) was provided by Shunshui Chemical Co., Ltd., Shanghai, China. Analytically pure sodium citrate, indomethacin, ethanol, sodium hydroxide, sodium chloride, acetic acid, hydrochloric acid, citric acid, and others were purchased from Shanghai Macklin Biochemical Co., Ltd., Shanghai, China.

The experimental equipment as well as their respective manufacturers used in this study were as follows: AL204-1C Electronic Balance manufactured by Mettler-Toledo Instruments (Shanghai, China) Co., Ltd.; Nicolet Nexus 6700 Fourier Transform Infrared Spectrometer manufactured by Shimadzu Technology (Shijiazhuang, China) Co., Ltd.; MARS40/60 Rotational Rheometer manufactured by Thermo Fisher Scientific (China) Co., Ltd. (Waltham, MA, USA); MicroMR02-025V 2 MHz Low-field Nuclear Magnetic Resonance Analyzer manufactured by Shanghai Newmai Electronic Technology Co., Ltd. (Shanghai, China); CTA-10HD Physical Property Analyzer manufactured by Tianjin Chuangxing Electronic Equipment Manufacturing Co., Ltd. (Tianjin, China); FOODBOT-D1 Extrusion 3D Printer manufactured by Hangzhou Shiying Technology Co., Ltd. (Hangzhou, China); RVA-TM Rapid Viscosity Analyzer manufactured by I-Xin Science and Technology (Beijing, China) Co., Ltd.; LC-16AAA Amino Acid Analyzer manufactured by Guangdong Shengze Technology Co., Ltd. (Dongguan, China); ARL EQUINOX 3000X X-ray Diffractometer manufactured by Shenzhen Keshida Electronic Technology Co., Ltd. (Shenzhen, China); and OS40-Pro Electronic Stirrer manufactured by Dalongxingchuang Laboratory Instruments (Beijing, China) Co., Ltd.

### 2.2. Determination of Amino Acid Composition in Lentinus edodes Protein

The amino acid composition was determined following the method described by Starzyńska-Janiszewska et al. [18]. The samples were placed in a 6 mol/L hydrochloric acid solution containing 0.5% phenol and incubated at 110 °C in an oven for 24 h. The resulting hydrolyzed products were dissolved in a sodium citrate buffer solution. Filtration was performed using a 0.45 μm syringe filter, and the amino acid composition was determined using an amino acid analyzer (Guangdong Shengze Technology Co., Ltd., Dongguan, China). The amino acids were eluted with a sodium citrate buffer solution gradient, derivatized with ninhydrin, and then measured using spectrophotometry at 570 nm and 440 nm.

Interventionary studies involving animals or humans, and other studies that require ethical approval, must list the authority that provided approval and the corresponding ethical approval code.

### 2.3. Preparation of LP/Potato Flour Mixture for 3D Printing

To prepare the material for 3D printing, 1% XG was added to 100 mL of deionized water and thoroughly mixed. LP was then mixed with potato flour at different ratios (0%, 10%, 20%, 30%, and 40%) until homogeneous. The mixture was poured into the gel solution at a ratio of 10:23 (powder to water) and the water volume was adjusted accordingly. The mixture was stirred using an electronic stirrer for 10 min and then heated in a 75 °C water bath for 20 min. After cooling to room temperature, the material was ready for subsequent 3D printing.

### 2.4. Three-Dimensional Printing of Microwaveable Food Products

An extrusion-based printer (Shiyin Tech. Co. Ltd., Hangzhou, China), equipped with an individual heating unit capable of controlling the printing temperature separately, was used to print simple line/pentagram and 3D structures of a cylinder (30 mm diameter and 18 mm height) to evaluate the printability of inks with different formulations and printing temperatures [19]. The 3D printing material prepared for the study was evenly filled into the 3D printing nozzle. The printing parameters were optimized through preliminary experiments as follows: a nozzle diameter of 1.2 mm, a printing speed of 15 mm/s, a linear infill pattern, 100% infill density, and 2 outer layers.

To evaluate the 3D printing characteristics of the material, a hollow cylinder with an outer diameter of 28 mm, an inner diameter of 18 mm, and a height of 15 mm was printed. Additionally, a cube with a side length of 15 mm was printed to assess the textural properties of the material. Furthermore, a star-shaped pattern with different infill densities (60%, 70%, 80%, 90%, and 100%) was designed for subsequent microwave treatments [6,20].

During the microwave treatment, a power level of 450 W was set, and treatment durations were set at 0, 1, 2, 3, and 4 min, respectively. After different microwave treatment durations, the length and height of the 3D-printed star-shaped samples were measured using calipers. The length was measured as the distance from one vertex to the opposite side, and the height was measured from the lowest point of the cut section to the bottom. The weight of each sample was measured using a balance, with three measurements taken for each sample [21].

### 2.5. Rheological Measurements

To measure the yield stress, the sample undergoes oscillatory stress scanning with the following parameter settings: a stress range of 1–1000 Pa at a frequency of 1 Hz. According to Liu Z et al. [6], a Rheometer (Thermo Fisher Scientific (China) Co., Ltd., Shanghai, China) was used to measure the dynamic viscoelasticity of different samples. The Rheometer employed a 40 mm geometry plate with a 1000 µm gap. The test procedure involved setting the temperature at 25 °C and applying a strain value of 0.1% within the angular frequency range of 0.1–100 rad/s. All tests were performed within the linear viscoelastic region.

### 2.6. Pasting Behaviors

To prepare the samples, different ratios of printing ink were mixed uniformly and poured into aluminum cans. Then, deionized water was added in a ratio of 3:1 (*w*/*v*) to the ink mixture. The gelatinization characteristics of the samples were determined using a rapid viscosity analyzer (RVA) (Thermo Fisher Scientific (China) Co., Ltd., Shanghai, China) following the method described by Wang et al. [22]. The testing procedure using the RVA was as follows: the sample was heated from room temperature (approximately 25 °C) to 95 °C at a rate of 5 °C/min, then held at 95 °C for 4 min. Subsequently, it was cooled to 50 °C at a rate of 5 °C/min and held for 4 min to obtain the gelatinization curves of different samples.

### 2.7. Texture Analysis

The upper firmness, filling softness, and base firmness of the samples were determined using an HDP/3PB probe (Tianjin Chuangxing Electronic Equipment Manufacturing Co., Ltd., Tianjin, China). The experimental parameters were set as follows: pre-test, mid-test, and post-test speeds of 2.5 mm/s, 2 mm/s, and 10 mm/s, respectively; compression strain of 45%; trigger force of 20 g; and a testing distance of 15 mm [6].

### 2.8. Low Field-Nuclear Magnetic Resonance (LF-NMR) Analysis

Water states and the mobility of ink with different formulations were analyzed by a LF-NMR analyzer (23.2 MHz, Numag Technology Co., Suzhou, China). The magnetic field strength was 0.5 T. During the test, about 2.5 g in sample was firstly packed using a thin layer of plastic film and then put into the NMR analyzer’s glass tube. The transverse relaxation time was measured by the Carr–Purcell–Meiboom–Gill (CPMG) sequence with the iteration times of 100,000 [23].

### 2.9. Fourier Transforms Infrared (FTIR) Spectroscopy

The ink samples were freeze-dried to remove moisture and then converted into a fine powder using a mortar. The powder was then mixed with KBr in a ratio of 1:100 (sample to KBr, *w*/*w*). The KBr pellet method, as described by Dankar et al. [24], was employed for sample preparation. A FTIR spectrometer (Thermo Co. Ltd., Fremont, CA, USA) was used for the analysis. The spectrometer recorded an average of 32 scans in the wavenumber range of 4000 to 400 cm^−1^, with a spectral resolution of 4 cm^−1^ at room temperature.

### 2.10. Sensory Evaluation

Twenty graduate students majoring in Food Science and Engineering were selected to evaluate the sensory quality of 3D-printed microwaveable food made from LP. A comprehensive scoring method was employed, and the scoring criteria can be found in Table 1. Prior to the experiment, the samples were randomly assigned identification numbers, and there was a specific time interval between evaluations of each sample. The evaluators were not allowed to communicate with each other during the evaluation process. To prevent flavor carryover, evaluators rinsed their mouths with water after tasting each sample. The evaluators were asked to use a scoring scale ranging from 1 to 10 to assess the following attributes of the samples: (1) moldability; (2) internal structure; (3) chewiness; (4) hardness; (5) crispiness; (6) aroma [25].

### 2.11. Statistical Analysis

Data analysis was conducted with SPSS (SPSS Statistics 17.0, SPSS Inc., Chicago, IL, USA); *p* values less than 0.05 with Duncan’s test were considered to be statistically significant. Origin 2018 (OriginLab Corp., Northampton, MA, USA) was used for graphing.

## 3. Results and Discussion

### 3.1. Analysis of LP Amino Acid Composition

The amino acid composition of LP is presented in Table 2, demonstrating its substantial abundance in 7 essential amino acids (EAA) and 10 non-essential amino acids (NEAA), with a total amino acid content (TAA) of 599.25 mg/g. The cumulative content of essential amino acids amounted to 258.45 mg/g, with leucine exhibiting the highest concentration at 54.25 mg/g. Similarly, the cumulative content of non-essential amino acids reached 340.8 mg/g, prominently featuring glutamic acid (63.86 mg/g), aspartic acid (56.33 mg/g), and proline (51.43 mg/g). The ratios of EAA to TAA and EAA to NEAA were 43.13% and 75.84%, respectively, aligning with the standards set forth by the Food and Agriculture Organization/World Health Organization (FAO/WHO) for an ideal protein. These findings underscored the substantial nutritional value and the high-quality protein reservoir provided by LP.

### 3.2. Rheological Properties and Correlation with 3D Printing Behavior

**Figure 1:**
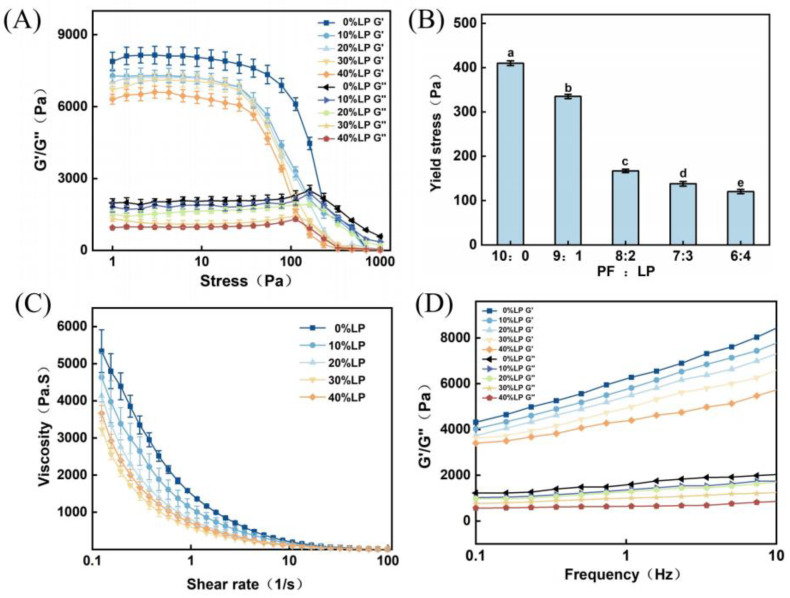
Rheological characteristic curves of 3D printing inks with different LP additions: (**A**) stress scanning; (**B**) yield stress; (**C**) shear viscosity curve; (**D**) viscoelasticity. Lowercase letters in the figure indicate statistical differences at *p* ≤ 0.05.

The rheological characteristic curves of 3D printing materials at various levels of Liquid Plasticizer addition are depicted in Figure 1. As shown in Figure 1a, in the stress sweep curve, the yield stress represents the shear stress necessary for initiating material flow. This parameter was commonly employed to assess the extrudability of the material during 3D printing. Previous studies have demonstrated that materials with lower yield stress were more favorable for extrusion [4,6]. Conversely, higher yield stress necessitated increased pressure to maintain normal extrusion of the material. Figure 1A reveals that as stress increases, the storage modulus (G′) of the material remains initially stable and subsequently declines rapidly, while the loss modulus (G″) remains stable, gradually increases, and then decreases. The point of intersection between G′ and G″ represents the yield stress.

Figure 1B showcases the yield stress of the 3D printing materials at different LP addition levels. It was evident that with increasing LP content, the yield stress of the material gradually decreased. In the absence of LP addition, the material exhibited the highest yield stress of 409.93 Pa. However, when the LP content reached 40%, the yield stress of the material decreased to 120.3 Pa. This finding indicated that the larger the amount of LP added, the lower the yield stress of the material. Increasing the LP concentration can effectively reduce the yield stress of the material, rendering it more amenable to normal extrusion. When the yield stress was low, the materials were more likely to extrude from the 3D printing tube.

Figure 1C illustrates the apparent viscosity curves of the 3D printing materials with different LP addition levels. Within the shear rate range of 0.1–1 s^−1^, all materials exhibited a rapid decrease in viscosity. As the shear rate increased to 1–10 s^−1^, the reduction in viscosity became more gradual. Beyond a shear rate of 10 s^−1^, the viscosity tended towards zero, indicating a characteristic shear-thinning behavior. With an increase in LP addition level, the viscosity of the material initially decreased and subsequently increased. The minimum shear viscosity was observed when the LP addition level reached 30%. However, when the LP content reached 40%, the viscosity of the 3D printing material started to rise. This phenomenon could be attributed to the formation of new hydrogen bonds between molecules due to the appropriate amount of LP. Under the influence of external shear forces, some hydrogen bonded between molecules breaks, resulting in a decrease in viscosity. However, as the LP addition level continued to increase, more entanglement occurred between the LP and potato starch molecules, leading to a denser structural arrangement and an increase in viscosity [26]. Moreover, it was worth noting that excessively high viscosity could impede the 3D printing process.

Figure 1D illustrates the viscoelasticity curves of the 3D printing materials with different LP addition levels. G′ and G″ characterize the self-supporting property of 3D printing raw materials. It could be observed that G′ exhibited a significant increase as the angular frequency increased, while G″ remained relatively unaffected by the angular frequency. Moreover, G′ was much greater than G″, indicating the predominant solid-like behavior of LP-based 3D printing materials [27].

### 3.3. The 3D Printing Performance Analysis of 3D-Printed Ink

**Figure 2:**
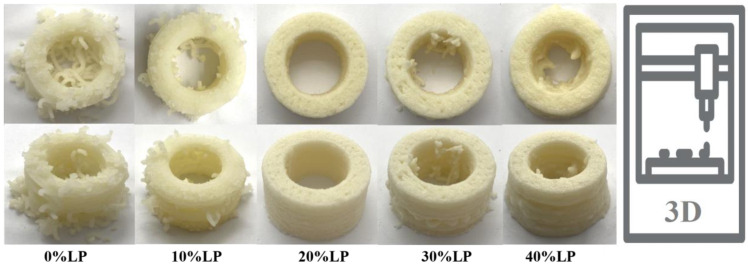
Effect of different LP addition on 3D printing property.

The printing effects of the 3D-printed ink incorporating varying levels of LP additions are depicted in Figure 2. It was evident that the performance of 3D printing exhibited an initial improvement followed by a subsequent deterioration as the LP content was increased. In the absence of LP, the potato starch powder underwent water absorption and swelling, resulting in an overall dry material composition. Consequently, the extrusion process during 3D printing became challenging, leading to frequent layer breaks and discontinuous printing. Upon introducing 10% LP, the material initially exhibited inadequate adhesion to the tray, and the structure at the bottom of the hollow cylinder appeared somewhat disordered. Although it could be printed in a normal manner, the surface of the cylinder was relatively rough. The addition of 20% LP facilitated a smoother printing process without any layer breaks or gaps. The resulting printed hollow cylinder exhibited a smooth and uniform surface. Upon reaching a 30% LP content, the material became slightly more diluted, yet it demonstrated improved self-supporting properties. However, internal layer breaks frequently occurred during the printing process. When the LP content reached 40%, the material became more diluted and viscous, leading to the collapse of the printed hollow cylinder and diminished self-supporting properties. Considering the rheological properties and 3D printing performance, further investigations should be conducted utilizing a 3D printing material containing 20% LP.

### 3.4. Comparative Analysis of 3D-Printed Samples and Molded Samples with Different Fill Ratios after Microwave Treatment

**Figure 3:**
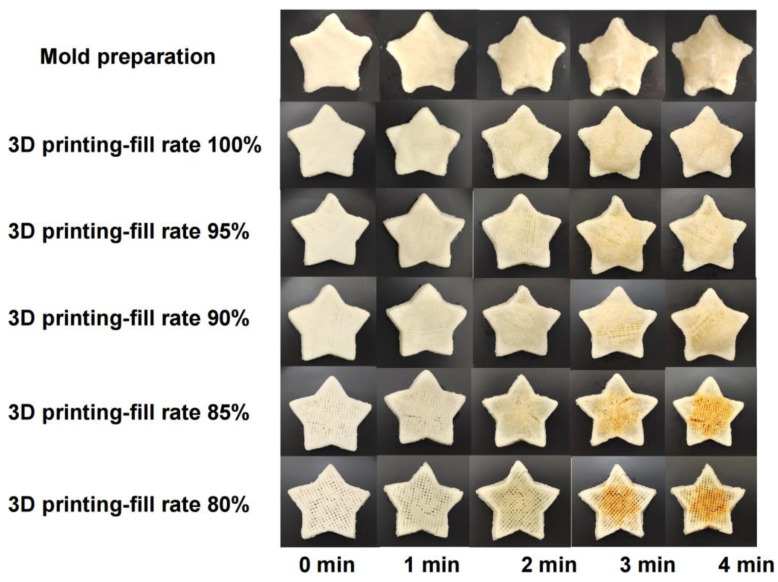
Comparative analysis of molded samples and 3D printing samples with different filling ratios after different microwave durations.

The visual appearance of the 3D-printed samples and molded extrusion samples with varying fill ratios following microwave treatment at different durations are presented in Figure 3. The surface of the 3D-printed samples exhibited uniform line structures, while the surface of the molded extrusion samples appeared relatively smooth. When the fill ratio was set at 100%, the surface of the samples gradually swelled as the microwave treatment duration increased. Particularly, when the microwave treatment durations exceeded 3 min, the surface of the samples experienced significant inflation. This phenomenon could be attributed to the higher fill ratio, which resulted in a uniformly compact internal structure within the samples. With prolonged heating, the molecular vibrations within the samples intensified, leading to enhanced internal expansion forces and accelerated swelling [28].

For fill ratios of 90% and 80%, the surface of the samples slightly depressed after 3 min of microwave treatment, yet the overall appearance and internal structure remained intact. Compared to the 100% fill ratio samples, this could be attributed to the rapid ventilation facilitated by the uniform small holes within the samples during heating, effectively preventing excessive swelling. However, when the fill ratio fell below 80%, the samples exhibited pronounced surface depressions with prolonged microwave treatment, and after 3 min, they began to turn yellowish. The reason for this might be that the samples with lower fill ratios had sparser internal line structures, rendering them more susceptible to collapse. Once heated, these samples struggled to withstand the expanding forces induced by the microwave, leading to internal depressions and intensifying the Maillard reaction.

The molded samples exhibited similarities to the 100% fill ratio printed samples. However, after 2 min of microwave treatment, the surface of the molded samples started to swell, and partial deformation occurred at the edges. In comparison, the 3D-printed samples experienced only proportional volume reduction, showcasing a clear advantage in maintaining their shape. Furthermore, 3D printing allowed for adjusting the fill ratio to modify the internal structure of the samples and achieved desired effects, an advantage that cannot be attained with molded extrusion samples.

**Figure 4:**
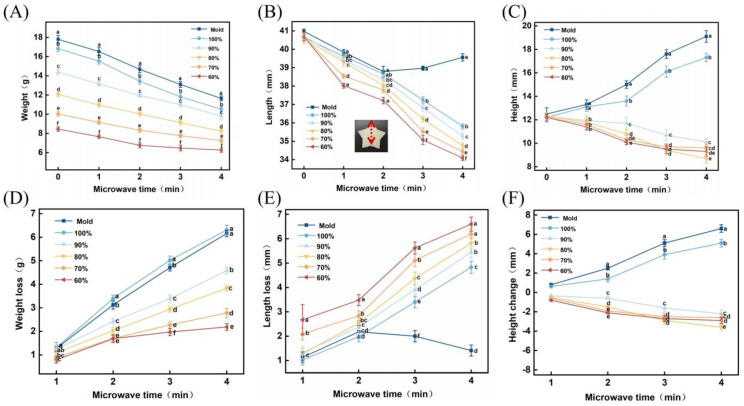
Comparative analysis of 3D-printed samples and molded samples with different fill ratios after microwave treatment. Changes in (**A**) weight, (**B**) length, and (**C**) height of mold samples and 3D printing samples with different filling ratios after different microwave duration; (**D**) weight loss, (**E**) length loss, and (**F**) height variation of molded samples and 3D printing samples with different filling ratios after different microwave durations. Lowercase letters in the figure indicate statistical differences at *p* ≤ 0.05.

Figure 4 presents the changes in weight, length, and height of the molded samples and 3D-printed samples with different fill ratios following varying durations of microwave treatment. Figure 4A reveals that samples with higher fill ratios exhibited greater weight. This could be attributed to the increased material required for filling in samples with higher fill ratios, resulting in a higher overall weight. As the microwave treatment durations increased, the weight of all samples decreased. Notably, the molded samples and samples with a 100% fill ratio demonstrated a more substantial weight reduction. This could be attributed to the rapid evaporation of moisture within the samples during microwave heating, facilitated by the rapid temperature rise. The larger surface area of samples with higher fill ratios led to more significant evaporation from the surface, resulting in a faster weight decrease.

Figure 4B reveals that the length of the 3D-printed samples gradually decreases; however, the molded samples exhibit an increase in length after 2 min of microwave treatment. This phenomenon might arise from the denaturation of proteins and the gelatinization of starch within the samples upon microwave heating, leading to overall sample shrinkage. However, the molded samples were more prone to deformation at the edges with prolonged microwave treatment, resulting in an increase in length.

Figure 4C reveals an upward trend in the height of the molded samples and samples with a 100% fill ratio, while the height of the other 3D-printed samples showed a slight decrease. This could be attributed to the increased molecular motion induced by heating, causing the samples to expand. However, samples with relatively smaller fill ratios possessed larger internal voids, allowing easier heat dissipation and resulting in a slower rate of expansion.

The changes in weight, length, and height of the 3D-printed samples and molded samples with different fill ratios following varying durations of microwave treatment are depicted in Figure 4. Among them, the samples with a 100% fill ratio and the molded samples exhibit the most pronounced changes in weight and height. These two samples possessed similar overall structures and demonstrated comparable trends. When the fill ratio in 3D printing was set below 100%, the weight loss of the samples decreased as the fill ratio decreased, while the length loss increased. Regarding height changes, the sample with a 90% fill ratio exhibited a smaller variation, while the other samples did not show significant differences. This phenomenon could be attributed to the samples with higher fill ratios possessing a more compact internal structure, making it easier for the samples to maintain their original structure. This finding indicated that adjusting the fill ratio inside the samples using 3D printing technology enabled the preservation of the spatial structure of the samples, and microwave treatment was proved to be a feasible post-processing method for preparing LP-based microwaveable food. Considering the observed changes in weight, length, and height of the samples, a fill ratio of 90% was chosen for further research on the product characteristics of 3D-printed samples.

### 3.5. Analysis of Gelatinization Characteristics of LP–Potato Starch

**Figure 5:**
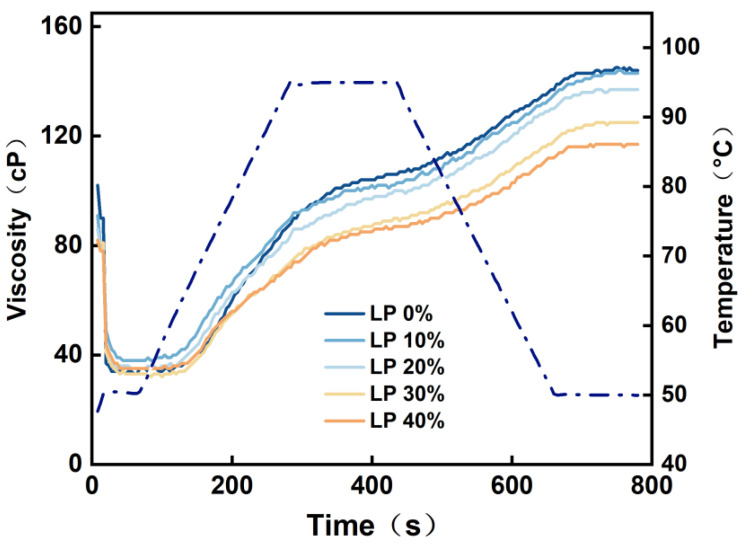
Gelatinization curve of potato starch with different concentrations of LP. The dashed line: the temperature curve of the sample over time.

The gelatinization curves of the mixed powder with varying levels of LP addition are depicted in Figure 5. The findings indicated a gradual decrease in viscosity as the concentration of LP increased. The viscosity curves of samples with LP concentrations below 30% were relatively close, while the reduction in viscosity became more pronounced when the LP concentration surpassed 20%.

The gelatinization parameters of the mixed powder with varying levels of LP addition can be found in Table 3. The findings demonstrated a gradual decrease in peak viscosity, trough viscosity, final viscosity, and setback value in the mixed powder as the content of LP increased. Specifically, the peak viscosity decreased from 106.47 cP to 87.26 cP, indicating that an elevated LP content reduced the viscosity of the mixed powder, subsequently diminishing the expansion ability of potato starch. This finding could be attributed to the LP coating the potato starch granules, thereby impeding their expansion in free water and leading to a reduction in paste viscosity (PV) within the material. Furthermore, the setback value decreased from 45.99 cP to 34.44 cP, indicating that the addition of LP influenced the stability of the cold paste within the mixed powder, making the potato starch less susceptible to aging. However, LP had no significant effect on the breakdown value, and the values were relatively low across all samples, signifying favorable thermal paste stability.

### 3.6. Analysis of Texture Characteristics of LP-Based 3D-Printed Microwave Food

**Figure 6:**
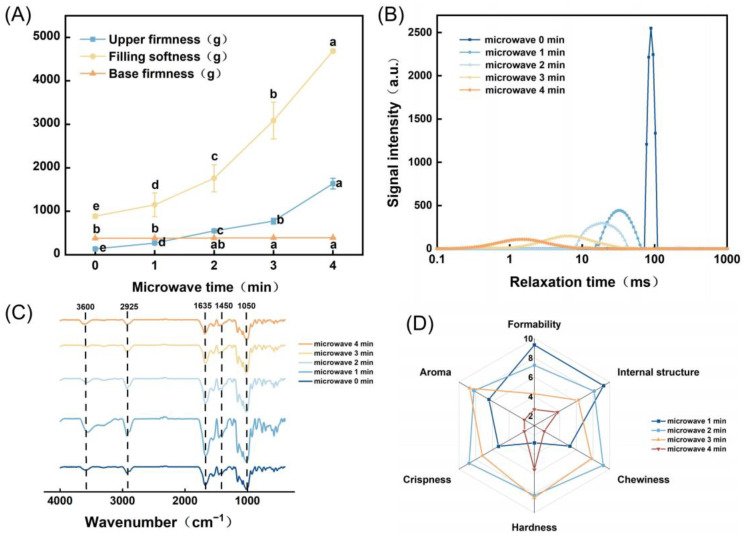
(**A**) Effect of different microwave durations on texture properties of LP-based 3D-printed food; (**B**) effect of different microwave durations on moisture distribution of LP-based 3D-printed food; (**C**) infrared absorption spectrum of LP-based 3D-printed food under different microwave times; (**D**) sensory evaluation of LP-based 3D-printed food under different microwave times. Lowercase letters in the figure indicate statistical differences at *p* ≤ 0.05.

The influence of different microwave durations on the texture characteristics of the LP-based 3D-printed food is depicted in Figure 6A. The findings revealed a progressive increase in surface hardness with prolonged microwave duration, while no significant change was observed in bottom hardness. Simultaneously, the internal softness gradually increased. This phenomenon could be attributed to the extended heating period, which led to rapid evaporation of surface moisture and heightened molecular activity within the sample. Consequently, the gelatinization of the potato starch occurred, resulting in a gradual enhancement of surface hardness and softening of the interior. Optimal surface hardness and internal softness of the LP-based 3D-printed food was achieved within the 2–3-mi microwave duration, yielding a crispy exterior and a tender interior texture.

### 3.7. Analysis of LF-NMR in LP-Based 3D-Printed Food

Figure 6B demonstrates the influence of different microwave durations on the moisture distribution in the LP-based 3D-printed food. The findings revealed that prior to microwave treatment, the samples exhibited a signal peak ranging from 77.53 to 102.34 ms, indicating the predominant presence of immobile water in the interior. As the microwave duration increased, the corresponding relaxation time of the signal peak gradually shifted towards the left. For samples treated for 1 min and 2 min, the peaks appeared at 15.70–62.95 ms and 8.41–41.50 ms, respectively. At these intervals, the interior largely retained immobile water. However, when the microwave duration reached 3 min and 4 min, the relaxation times for the samples were 2.64–58.73 ms and 0.03–14.65 ms, respectively. This finding suggested the coexistence of bound water and immobile water within the samples after 3 min of microwave treatment. Upon reaching 4 min, the interior of the samples primarily consisted of bound water, indicating a significant transition from immobile water to bound water in the moisture distribution within the LP-based 3D-printed food during the entire microwave process. The proteins and starch molecules in the samples were tightly bound to water molecules, primarily attributed to the elevated temperature resulting from microwave heating, leading to shrinkage and alterations in the internal moisture content.

### 3.8. FT-IR Analysis of LP-Based 3D-Printed Food

The infrared absorption spectra of the LP-based 3D-printed food under different microwave durations are depicted in Figure 6C. Infrared spectroscopy was the primary method used to detect the presence of hydrogen bonds and assess their strength. Lower wave numbers indicated stronger interactions between molecules. The graph reveals the characteristic peaks at wavelengths of 3600, 2925, 1635, 1450, and 1050 cm^−1^. As the microwave duration increased, no new characteristic peaks emerge, suggesting that microwave treatment might not induce changes in the chemical structure of the samples to generate new functional groups.

According to the results of previous research, the characteristic peak around 1050 cm^−1^ was attributed to the bending vibration absorption of monosaccharide and polysaccharide hydroxyl groups, caused by the stretching vibration of C-O bonds and the bending vibration of C-H in potato starch. The peak at 1450 cm^−1^ was attributed to the bending vibration of CH_3_ groups. The peak at 1635 cm^−1^ was associated with the stretching vibration of C=O bonds. The peak at 2925 cm^−1^ was caused by the asymmetric stretching vibration of CH_2_ groups. The peak at 3600 cm^−1^ was attributed to the OH bond.

These findings indicated the presence of specific functional groups in the LP-based 3D-printed food. In addition, microwave treatment did not induce the formation of new functional groups or alter the chemical structure of the samples [29].

### 3.9. Sensory Evaluation Analysis of LP-Based 3D-Printed Food

The sensory evaluation of the LP-based 3D-printed food under various microwave durations is presented in Figure 6D. The results demonstrated that as the microwave duration increased, the scores for moldability and internal structure of the samples gradually decreased. On the other hand, the scores for aroma, crispiness, texture, and chewiness initially increased and then decreased.

The product microwaved for 1 min exhibited minimal deformation, a fine and regular cross-section, and received high scores for moldability (9.333) and internal structure (9.256). However, the texture was relatively soft, and the chewing sensation was average, with an average score of 6.382. The product microwaved for 2 min showed a slightly swollen surface, a regular cross-section, moderate softness, crispy exterior, and tender interior. It received the highest scores for chewing sensation (9.213) and crispiness (8.777). The aroma emitted from microwave heating was also more pronounced, with an average score of 8.295. The product microwaved for 3 min had a moderate texture, a strong aroma, but due to slightly longer microwave duration, the surface was slightly depressed, and the cross-section was prone to damage. The internal structure and moldability received poorer scores, with an average score of 7.121. The product microwaved for 4 min exhibited severe deformation on the surface, an irregular cross-section, significant damage, overall hardness, poor chewing sensation, and a burnt taste due to prolonged heating. The average score for all indicators was only 3.091. Considering all the indicators, the LP-based 3D-printed food microwaved for 2 min received the highest overall score, with an average score of 8.295, indicating that it was highly favored by consumers.

## 4. Conclusions

This study focused on the development of 3D-printed microwaveable food using LP, which is a high-quality protein source with a balanced amino acid composition. The researchers utilized LP and potato flour in their formulation and examined the characteristics of the resulting product. A rheological analysis revealed that as the LP content increased, the yield stress and viscoelasticity of the printing material gradually decreased, while the shear viscosity initially decreased and then increased. Based on the 3D printing characteristics and rheological results, the optimal 3D printing formulation was determined to be LP: potato flour: xanthan gum: water = 2:8:1:23. A comparison was made between 3D-printed samples and molded samples with different filling ratios. After microwave treatment, the 3D-printed samples exhibited better shape, weight, and size. The study found that a filling ratio of 90% resulted in the best microwave effect for the product. The analysis of product characteristics showed that as the LP content increased, the viscosity and retrogradation value of the shiitake mushroom–potato starch mixed powder gradually decreased, while the decay value showed no significant change. With increasing microwave time, the top surface of the product became harder, the interior became softer, and the hardness of the bottom remained unchanged. The moisture inside the product transformed from immobile water to bound water. The highest overall score for the LP-based 3D-printed food was achieved when it was microwave processed for 2 min, reaching 8.295 points.

Edible mushroom protein holds significant developmental potential; however, there is currently limited research on the processing characteristics of such protein. Our team has previously explored the feasibility of employing 3D printing technology to create black fungus food suitable for individuals with chewing and swallowing difficulties [30]. Moreover, our findings indicate that the *Pleurotus ostreatus* protein not only enhances the viscosity and consistency index of 3D printing ink but also bolsters the mechanical strength of printed structures, demonstrating strong suitability for 3D printing applications [6]. This study significantly advances the potential of 3D-printed food utilizing the LP protein. It offers insights into formulation, printing parameters, and microwave processing, showcasing the viability of creating microwaveable food with appealing sensory attributes. The optimized formulation and filling ratio establish a framework for desired internal structure, while insights into microwave treatment’s impact on physical properties pave the way for customized and nutritious food products. This research contributes to sustainable and personalized food manufacturing, fostering innovation in 3D food printing technology.

## Figures and Tables

**Table 1:** Sensory evaluation standards of LP in microwave food.

Score	Moldability	Internal Structure	Chewiness	Hardness	Crispiness	Aroma
7–10	Good moldability	Uniform, fine, and regular cross-section	Good chewiness	Moderate hardness	Crispy on the outside, tender on the inside	Strong aroma
4–6	Average moldability	Partially broken cross-section	Average chewiness	Hard or mushy	Average tenderness	Slight aroma
0–3	Poor moldability	Irregular cross-section	Poor chewiness	Too hard or too mushy	Poor tenderness	Burnt taste

**Table 2:** Analysis of amino acid composition of LP.

Essential Amino Acids (EAA)		Non-Essential Amino Acids (NEAA)	
Category	Amino Acid Content (mg/g)	Category	Amino Acid Content (mg/g)
Threonine (Thr)	28.63	Aspartic Acid (Asp)	56.33
Valine (Val)	41.17	Serine (Ser)	30.15
Methionine (Met)	28.24	Glutamic Acid (Glu)	63.86
Isoleucine (Ile)	40.77	Glycine (Gly)	27.17
Leucine (Leu)	54.25	Alanine (Ala)	32.49
Phenylalanine (Phe)	28.13	Cysteine (Cys)	3.27
Lysine (Lys)	37.26	Tyrosine (Tyr)	27.01
		Proline (Pro)	51.43
		Arginine (Arg)	32.64
		Histidine (His)	16.45
Total	258.45	Total	340.8
EAA/NEAA	75.84%	EAA/Total Amino Acids (TAA)	43.13%

**Table 3:** Gelatinization parameters of potato starch with different concentrations of LP.

Sample	Peak Viscosity (cP)	Trough Viscosity (cP)	Final Viscosity (cP)	Breakdown Value (cP)	Setback Value (cP)
0%	106.47 ± 2.31 ^a^	102.63 ± 3.16 ^a^	148.62 ± 3.16 ^a^	3.84 ± 0.25 ^a^	45.99 ± 0.83 ^a^
10%	102.79 ± 3.27 ^b^	100.71 ± 1.64 ^b^	143.84 ± 2.89 ^b^	2.08 ± 0.21 ^c^	43.13 ± 0.45 ^b^
20%	98.24 ± 2.95 ^c^	95.62 ± 1.93 ^c^	137.65 ± 3.07 ^c^	2.62 ± 0.37 ^b^	42.03 ± 0.72 ^c^
30%	90.16 ± 1.85 ^d^	87.99 ± 2.56 ^d^	125.24 ± 2.34 ^d^	2.17 ± 0.28 ^b^	37.25 ± 0.64 ^d^
40%	87.26 ± 2.17 ^e^	83.37 ± 2.83 ^e^	117.81 ± 3.15 ^e^	3.89 ± 0.43 ^a^	34.44 ± 0.51 ^e^

Note: the first column represents the amount of LP added, and each value represents the average ± standard deviation (*p* ≤ 0.05). Different low case letters above columns indicate statistical differences at *p* ≤ 0.05.

## Data Availability

Data available on request from the authors.
